# Filamentous Influenza Viruses

**DOI:** 10.1007/s40588-016-0041-7

**Published:** 2016-07-02

**Authors:** Matthew D. Badham, Jeremy S. Rossman

**Affiliations:** 1 School of Biosciences, University of Kent, Canterbury, Kent CT2 7NJ, UK

**Keywords:** Influenza virus, Morphology, Filaments, Assembly, Budding

## Abstract

Influenza A virus is a pathogen of global medical importance causing significant health and socio-economic costs every year. Influenza virus is an unusual pathogen in that it is pleomorphic, capable of forming virions ranging in shape from spherical to filamentous. Despite decades of research on the influenza virus, much remains unknown about the formation of filamentous influenza viruses and their role in the viral replication cycle. Here, we discuss what is known about influenza virus assembly and budding, focusing on the viral and host factors that are involved in the determination of viral morphology. Whilst the biological function of the filamentous morphology remains unknown, recent results suggest a role in facilitating viral spread in vivo. We discuss these results and speculate on the consequences of viral morphology during influenza virus infection of the human respiratory tract.

## Introduction

Between 1918 and 1920, an estimated 50–100 million people died from the first recorded pandemic of a viral infection commonly known as the flu [1]. This outbreak, nowadays known as the Spanish flu, was caused by the Influenza A virus (IAV). Since 1920, IAV has continued to cause pandemics with the most recent being the 2009 H1N1 swine flu pandemic [2] which was estimated to have infected over 60 million people and caused 12,000 deaths in the USA alone [3].

IAV is a pleomorphic virus of the Orthomyxoviridae family consisting of a segmented, single stranded, negative sense RNA genome [4]. Responsible for seasonal outbreaks and occasional pandemics IAV is a major burden on health systems globally and is estimated to cost the US economy $87.1bn annually [5]. Five to 15 % of the Northern hemisphere’s population is estimated to be affected per annum [6] with 250,000–500,000 deaths globally per year [7]. The most severe illness occurs in immuno-compromised, elderly and very young individuals and is often followed by secondary bacterial pneumonia, resulting in significant morbidity and mortality.

IAV infects the cells of the upper respiratory tract, causing illness in a wide range of hosts, including humans, pigs, horses and birds. Birds are thought to be the reservoir for IAV in the wild [8] and are a key source for the emergence of novel IAV strains [9], such as the 1918 Spanish flu. Recent strains emerging from the wild bird population include the high pathogenicity avian influenza virus strains H5N1 and H7N9, currently circulating in Eastern Asia where they have up to a 60 % case-fatality rate in humans, though do not yet spread efficiently from person to person [10].

## The Influenza A Virus

The IAV genome comprises of 11 genes on eight RNA segments. These can broadly be categorised as encoding the viral structural or non-structural proteins. Haemagglutinin (HA), neuraminidase (NA), nucleoprotein (NP) and matrix proteins one and two (M1 and M2) are primarily structural proteins with additional functional roles, whilst the polymerase subunits (PB1, PB2 and PA) and the non-structural proteins (NS1 and NS2) serve mainly function roles during virus replication. NP binds to and is involved in the packaging of the viral genome along with the polymerase subunits for transport and assembly [11] and also interacts with various cellular proteins, such as CRM1 involved in nuclear export of the replicated viral genome [12]. HA is responsible for virus attachment to a target cell by recognition of sialic acid residues on the cell surface [13]. Following attachment, the virus undergoes receptor-mediated endocytosis and subsequent endosomal acidification triggers HA fusion activity wherein HA mediates the fusion of the viral envelope with the endosomal membrane, freeing the viral genome to traffic to the nucleus [14]. NA has enzymatic activity, cleaving sialic acid bonds and releasing newly formed viruses from the host cell [15–17]. NA is the target of small molecule pharmaceuticals used to treat influenza: Relenza® (zanamivir) and Tamiflu® (oseltamivir) [18]. These are structural analogues of sialic acid, and work to inhibit the enzymatic action of NA, thus retaining newly formed virus on the host cells. HA and NA are viral surface proteins and the main antigenic determinants of the virus. Antigenic variations in HA and NA give rise to the nomenclature ‘H’ and ‘N’ (e.g. H1N1). The matrix proteins, M1 and M2, are formed from alternative reading frames of RNA segment seven [19]. M1 is the most abundant viral protein forming a scaffold beneath the host membrane derived viral envelope. M1 anchors NA, HA and M2 in place within the envelope and interacts with the viral genome in the ribonucleoprotein (RNP) complex. M2 is a transmembrane ion channel protein, which plays a fundamental role in the initial stages of viral infection. Once in the acidic endosome, M2 allows protons to enter the virus, triggering uncoating [20] and the release of the viral RNP from M1 [21]. M2 also plays a pivotal role in IAV budding by altering membrane curvature, facilitating the assembly of filamentous virions and then mediating membrane scission and the release of budding viruses [22].

## Viral Assembly and Budding

It is thought that IAVassembly and budding occurs at lipid raft domains on the apical surface of the host cell plasma membrane, where IAV proteins are brought together in high concentrations within specific membrane regions [23]. NA and HA are both fundamentally associated with these domains, with the transmembrane domain of HA promoting the raft association [24]. HA has the ability to induce budding of virus-like particles (VLPs) in and of itself, forming vesicles similar in appearance to viruses [25]. This suggests that HA may possess an intrinsic capacity to alter membrane curvature. Alternatively, the induction of membrane curvature may be driven by the crowding of HA molecules within a defined space (i.e. within a lipid raft domain). However, HA VLP budding only produces spherical particles, whereas VLPs are filamentous when M1 is also expressed [26••]. After the formation of a viral bud, the virus remains attached to the host cell through a small membrane neck. At this point, the M2 protein alters membrane curvature, constricting the neck and causing membrane scission [27]. The enzymatic action of NA can then release the fully formed virus from the host cell. Throughout this process, it is not clear when the genome is recruited to the budding virion nor the effects RNP binding has on the budding process or the formation of filamentous virions.

## Viral Morphology

**Fig. 1:**
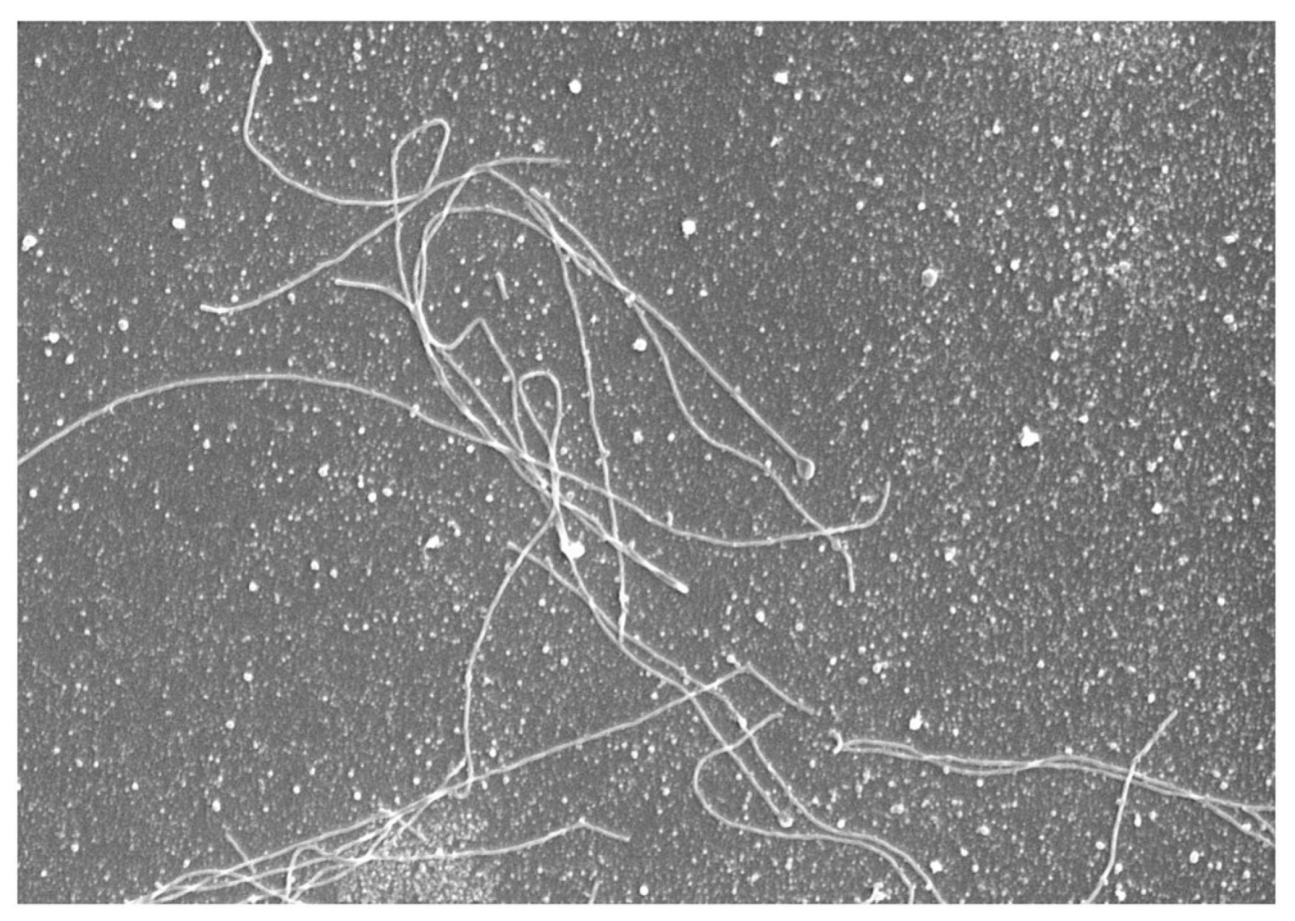
Filamentous and spherical influenza virions. MDCK cells were infected with 3 MOI of A/Udorn/72 influenza virus for 18 h. The supernatant was harvested, fixed and processed for scanning electron microscopy. Image is 14 × 20 μm

IAV is a pleomorphic virus, known to display a range of morphological states, from filamentous to spherical, with ovoid or bacilliform intermediates often observed (Fig. 1). In certain cases, IAV strains may produce solely spherical virions; however, filament-producing strains always produce a mixture of both filamentous and spherical virions. It is known that filamentous viruses contain only one copy of the IAV genome, thus each sphere, bacilliform or filament is thought to be a single infectious unit regardless of length. Structurally, viral filaments are roughly equal, or slightly smaller in diameter (80–100 nm) to spherical virus (120 nm), but extend to a significant length, sometimes upwards of 20 μm, with lengths over 50 μm not unheard of. Filamentous viruses are particularly of note as they are recurrently observed in human clinical infections [28–34, 35••, 36] (for example, filamentous virions are seen in lung sections from fatal cases of the 2009 H1N1 pandemic [37]). In contrast, many laboratory strains produce solely spherical virions. The biological function of this morphology is not known nor is it understood how host adaptation can select for a specific viral morphology; however, repeated passaging of filamentous human clinical isolates in chicken eggs causes a morphological adaptation resulting in the production of only spherical virus [28, 38] whereas adaptation to growth in guinea pigs restores filament formation [35••]. Mutations of several different viral proteins can influence filament formation during the process of adaptation. For example, the filamentous A/Udorn/72 strain becomes spherical with a single point mutation in the M1 protein [39–41]. Thus, a range of both host and viral factors governs the formation of filamentous virions during influenza virus assembly and budding.

## Viral Determinants of Morphology

Many different studies have investigated the viral factors that determine morphology, with most focusing on the structural proteins M1 and M2 and their role in viral assembly. M1 plays a crucial role in the assembly and budding of both filamentous and spherical IAV [23, 24]. In 2004, Elleman and Barclay reported that M1 was also the main viral determinant of filamentous morphology [42]. Swapping the ‘M’ RNA segment from the spherical strain, A/Puerto Rico/8/1934 (PR8), with the M segment of the filamentous strain, A/Udorn/1972 (Udorn), enabled the conversion of a spherical strain into a filamentous strain (when M-Udorn was inserted into PR8) and a filamentous strain into a spherical strain (M-PR8 into Udorn) [23, 40]. In 2007, Chen et al. showed that M1 is required for the formation of filamentous VLPs, though it was not required to form bacilliform (<1 μm) or spherical VLPs [25, 43, 44].

Interestingly, in filamentous viruses, M1 appears to adopt a helical conformation [14], which is not apparent in spherical virus, suggesting that structural variations in the M1 protein may govern viral structure [45]. As the most abundant viral protein, M1 forms a layer under the viral envelope and is responsible for interacting with NA, HA and M2 to form a scaffold-like complex [46]. It is postulated that M2 can stabilise this complex during budding to allow for continued M1 polymerisation and the formation of a viral filament [22, 47]. In support of this hypothesis, it has been observed that mutation of the M2 protein can dramatically affect viral morphology, with mutations in the c-terminal amphipathic helix converting a filamentous virus into a spherical one [47], whereas truncation of the c-terminus at residue 70 enables filament formation from an otherwise spherical virus [48]. The effect of M2 on viral filament formation has also been studied using the monoclonal antibody 14C2, which has been shown to inhibit filamentous virus formation whilst permitting spherical virus to bud [47]. 14C2 binds to the ectodomain of M2 and is thought to disrupt the binding between the M2 cytoplasmic tail and M1 [47]. This is consistent with other data showing that mutation of the M2 cytoplasmic tail between residues 70–77 reduces M1-M2 interactions and subsequently the amount of M1 and RNP packaged in virions [49, 50]. Intriguingly, a single amino acid substitution in the M2 cytoplasmic tail of the filamentous Udorn strain, Y76A, significantly reduced the number and length of filamentous viruses produced [50]. However, recovery of the filamentous morphology was seen with the addition of a S71Y mutation, though it is not clear if these mutations also affect M1-M2 interactions [50]. In either case, it is possible that the M2 protein affects viral filament formation by altering membrane curvature, stabilising the site of budding and therefore enabling M1 polymerisation and the elongation of a viral filament [18, 20]. Thus, M2 appears to modify filament formation through binding and recruitment of M1, whereas M1 itself is required for the actual structuring of the filament. This suggests that filamentous virion production is likely a multifaceted process, affected by several different viral proteins, all occurring in the context of an array of cellular partners.

## Host Determinants of Morphology

IAV is pleomorphic, adopting both spherical and filamentous forms [45]. As described, viral morphology can be altered through adaptation to different hosts, implying that there are host-specific influences on filament formation [35••]. Filamentous IAV is consistently found in human clinical isolates from laboratory confirmed cases, in the 2009 H1N1 pandemic [37] and as far back as the 1957–1958 pandemic [38], with the first identification having occurred in 1946 [51]. It is known that filament forming strains become spherical after repeated passage in embryonated chicken eggs, whereas the filamentous morphology is retained during passage in Madin-Darby canine kidney (MDCK) cells [28, 35••, 52]. Thus, host cell factors play a considerable role in filament formation, and IAV morphology may represent an adaptation to a specific host cell environment. Previous research has identified several host proteins that affect viral morphology. In particular, when the Udorn virus is grown in polarised MDCK cells, filamentous virus is produced from the apical plasma membrane [23, 24, 45]. Chemical disruption of the actin cytoskeleton causes depolarization of the cells and specifically reduces filamentous virus production whilst having no effect on the budding of spherical virus [53]. Considering that the upper respiratory tract consists of highly polarised epithelial cells and is the primary site of human infection, the detection of filamentous IAV in human clinical samples may be directly related to epithelial cell polarisation. However, other experiments have shown that human embryonic kidney 293T cells are capable of producing filamentous virions, despite an absence of cell polarisation and a lack of a defined apical membrane [54]. Thus, the impact of cell polarisation on viral morphology may be more complicated and may be influenced by other host cell proteins or processes.

In 2010, Bruce et al. reported that Rab11 and Rab11-family interacting protein 3 (FIP3) are necessary for the formation of filamentous virus [55]. Rab11 is a GTPase involved in the recycling endosome pathway and plays a role in protein and vesicle transport [56]. Rab11 is necessary for the budding of all morphologies of IAV [54]; however, Rab11-FIP3 is essential for the formation of filamentous IAVs [55]. The FIPs regulate localisation and trafficking of target proteins [57] by interacting with motor proteins, such as myosin Vb [58] and kinesin [59]. FIP3 is involved in the regulation of Rab11 and, subsequently, in several cellular pathways, such as the essential trafficking of endosomes to the cleavage furrow during cytokinesis [60] and the regulation of actin filaments [57]. The polymerase subunit PB2 of the viral RNP complex interacts with Rab11, which is complexed with cellular transport vesicles and FIP3 [61, 62•]. RNPs then essentially ‘piggyback’ on these vesicles, travelling with Rab11 from the MTOC to the site of virus budding along the microtubule network [63]. Once near the plasma membrane, these RNP-Rab11-vesicle complexes uncouple in an uncharacterized mechanism, alluded to by the fact that budded virus (both spherical and filamentous) are devoid of Rab11 [64]. It is speculated that interactions with M1 are then responsible for organisation of RNPs at the plasma membrane, as M1 is intrinsically associated with RNPs in late stage infection to prevent their re-entry in to the nucleus [65]. It has not yet been demonstrated, however, if the association with M1 uncouples the Rab11-RNP complexes or if Rab11-transported vesicles contribute any additional components to the budding virus. One possible Rab11-associated component is FIP3, though how this protein contributes to viral filament formation remains unclear. However, it is apparent that the morphology of IAV depends on both cellular and viral factors.

## Functions of Viral Morphology

The biological significance of IAV morphology in human clinical infections is a subject of great interest. The production of viral filaments appears to be highly inefficient by its nature, consuming anywhere from three to thirty times the amount of plasma membrane used to bud one infectious virus [51, 53]. There are several opinions on why, despite this apparent inefficiency, IAV readily produces filamentous virus in human clinical infections. As there is always a mixed population of spheres and filaments and never solely filaments, it is possible that the two morphologies are playing different roles within the host. It has been found that filament forming mutants of PR8 have higher per-molecule NA activity in vitro [66••]. In addition, NA has been shown to cleave sialic acid bonds within the mucus secreted by airway epithelial cells [67, 68] and the greater number of NA molecules (owing to a longer viral length) may serve to more efficiently clear this mucus layer. It is therefore plausible to think that the filamentous morphology is actually a marker of pathogenicity in vivo, whereby mucus in the airway is cleared by NA on filaments, thus allowing for a more efficient spread of the smaller spherical viruses [69]. This hypothesis is supported by a recent study that suggests that filaments are not released as efficiently as spheres from cells and may remain as cell-associated virions [44].

In 1998, it was shown that spherical and filamentous viruses are comparably infectious in vitro and both contain a single copy of the viral genome [53, 69–71]. However, it has recently been reported that certain subsets of filamentous virions may lack a genome [69]. Vijayakrishnan et al. (2013) reported that longer filaments were typically devoid of a copy of the viral genome, whereas shorter filaments were not. Thus, there might not be a single type of filamentous virions, but rather a range, potentially with different functions. In the tightly packed epithelial layer of the upper respiratory tract, short, cell-anchored, infectious filaments may be able to directly deliver the viral genome to neighbouring cells without the need to release and transmit a viral particle. This process may be facilitated by the more permissive use of macropinocytosis as an alternate cell entry pathway, used by filamentous IAV [72–74]. At the same time, longer, non-infectious filamentous virions may serve to thin and clear host respiratory mucus, facilitating the spread of spherical virions to neighbouring cells and to new hosts.

## Conclusions

In this review, we have explored recent findings in how IAV is assembled and budded and how some of these mechanisms, especially from a cellular aspect, may influence the budding of filamentous IAV. Whilst many studies have found an effect of cellular proteins and viral protein sequences on filament formation, the causation and function of filamentous IAV remains to be fully understood. What recent data shows for certain is that IAV morphology is not solely cell or virus dependent but relies on a precise balance and interaction between the two. A single point mutation in a viral protein can alter morphology whereas silencing of a single cellular gene can also affect the ability of an IAV strain to form filamentous virions. Furthermore, a strain that forms filamentous virions in vivo will not necessarily do so in vitro or in a different host species. Ultimately, the role of filamentous IAV in human infection remains an enigma. Given the resource requirements needed to produce a single filamentous IAV as compared to a single spherical virion, it is likely that the filamentous morphology confers some advantage to the virus. Future investigations of IAV host-pathogen interactions will undoubtedly shed more light on the mechanisms of viral morphogenesis, allowing for a better understanding of the mechanisms of influenza virus budding, the functions of viral morphology and the impact of morphology on influenza disease in human clinical infections.
